# Transcriptomic Analyses Reveal Long Non-Coding RNA in Peripheral Blood Mononuclear Cells as a Novel Biomarker for Diagnosis and Prognosis of Hepatocellular Carcinoma

**DOI:** 10.3390/ijms23147882

**Published:** 2022-07-17

**Authors:** Pattapon Kunadirek, Nutcha Pinjaroen, Intawat Nookaew, Pisit Tangkijvanich, Natthaya Chuaypen

**Affiliations:** 1 Center of Excellence in Hepatitis and Liver Cancer, Department of Biochemistry, Faculty of Medicine, Chulalongkorn University, Bangkok 10330, Thailand; pkunadirek@gmail.com; 2 Department of Radiology, Faculty of Medicine, Chulalongkorn University, Bangkok 10330, Thailand; fon_nutcha@hotmail.com; 3 Department of Biomedical Informatics, College of Medicine, University of Arkansas for Medical Sciences, Little Rock, AR 72205, USA; inookaew@uams.edu

**Keywords:** hepatocellular carcinoma, peripheral blood mononuclear cells, transcriptomic profile, long non-coding RNA, biomarker, alpha-fetoprotein

## Abstract

Novel biomarkers are highly required for the diagnosis and predicting prognosis of hepatocellular carcinoma (HCC). In this study, we investigated the profiles of long non-coding RNAs (lncRNAs) obtained from the peripheral blood mononuclear cells (PBMCs) of patients with HCC and PBMCs from a co-culture model using transcriptomic analysis. The differentially expressed lncRNAs (DElncRNAs) were then characterized and integrated as cancer-induced lncRNAs. Among them, three up-regulating DElncRNAs including *MIR4435-2HG*, *SNHG9* and *lnc-LCP2-1* and one down-regulating, *lnc-POLD3-2*, were identified. The functional analysis showed that these enriched lncRNAs were mainly associated with carcinogenesis and immune responses. Following further validation in PBMCs samples (100 HBV-related HCC, 100 chronic hepatitis B and 100 healthy controls), *MIR4435-2HG*, *lnc-POLD3-2* and their combination were revealed to be sensitive biomarkers in discriminating HCC from non-HCC (AUROC = 0.78, 0.80, and 0.87, respectively), particularly among individuals with normal serum alpha-fetoprotein levels. Additionally, high circulating *SNHG9* expression was shown to be an independent prognostic factor of overall survival in patients with HCC. These results indicate that determining these lncRNAs in PBMCs could serve as novel diagnostic and prognostic biomarkers for HBV-related HCC.

## 1. Introduction

Hepatocellular carcinoma (HCC) is the sixth common cancer type with approximately 250,000 estimated new cases for each year and is the third leading cause of cancer-related motility worldwide [1]. Chronic hepatitis B virus (HBV) infection is considered as the common risk factor of HCC worldwide, especially in East and Southeast Asia [2]. Most of HCC patients are usually diagnosed at the intermediate or advanced stage, for which the curative treatments are limited [2]. It is urgently important to access the HCC at the initial stage for improving the HCC surveillance. Although serum alpha-fetoprotein (AFP) has been widely used as a biomarker for HCC screening, the sensitivity of AFP (58–68%) in detecting early-stage of HCC is still not satisfactory [3]. In addition, the elevation of serum AFP may be found in patients with non-malignant chronic liver disease, such as chronic hepatitis infection and cirrhosis [3]. Therefore, identifying novel biomarkers for early diagnosis and prognosis are highly required to improve the health care outcomes and survival in HCC patients. Recently, we demonstrated that the alteration of mRNAs in HCC-induced peripheral blood mononuclear cells (PBMCs) in a co-culture model represented cancer-induced genes that could be used as a novel diagnostic and prognostic marker for HCC [4]. However, the regulation of lncRNAs in PBMCs has not been elucidated in HCC.

Long non-coding RNAs (lncRNAs) are a subtype of ncRNA molecules, which are defined as having a length longer than 200 nucleotides and are not translated into protein [5]. They have functioned as regulatory molecules in many biological processes, such as cell proliferation, apoptosis, and invasion due to their ability to act as signals, guides, scaffolds, or decoys of other RNA molecules [5]. Several studies suggested that the aberrant expression of lncRNAs is linked to tumor development and metastasis in many types of cancers, including HCC [6,7]. For instance, the overexpression of *HOTAIR* negatively regulated miR-1 and promoted HCC progression [8]. The alteration of lncRNAs is also involved in immune response in cancers, including the regulation of immune checkpoint expression and tumor microenvironment composition [9]. Regarding HCC, it was shown that an up-regulated lncRNAs signature was associated with increased immune infiltration and worse overall survival [10].

In this study, the differentially expressed long non-coding RNAs (DElncRNAs) obtained from the co-culture model and PBMCs of patients with HCC were identified using RNA-sequencing and bioinformatic analysis tools. The lncRNA–miRNA–mRNA regulatory network was also constructed according to competing endogenous RNA (ceRNA) networks [11]. Finally, the intersect lncRNAs were characterized as putative novel biomarkers and were further validated in PBMCs from patients with HBV-related HCC.

## 2. Results

### 2.1. Identification of lncRNA Candidate Biomarkers in PBMCs

**Figure 1:**
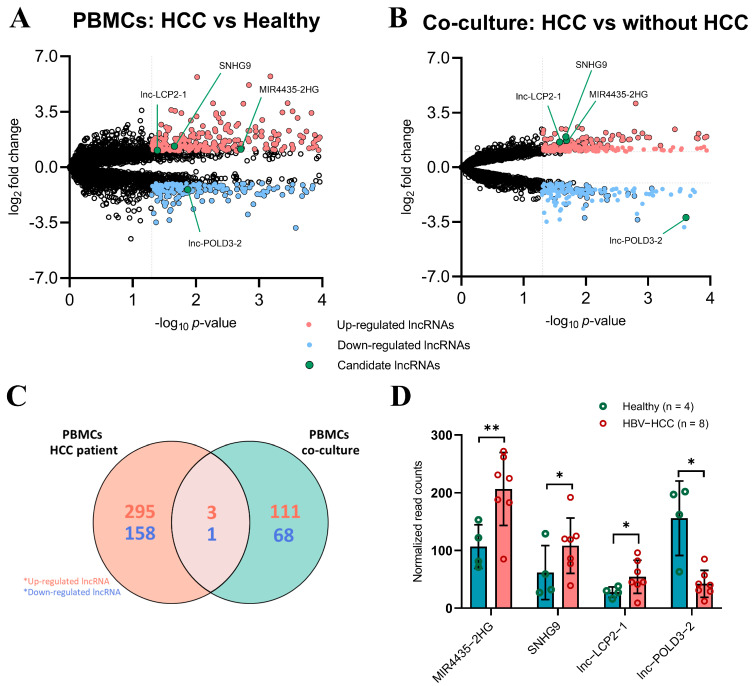
Transcriptomic profile of cancer-induced lncRNAs and integration transcription profiles of PBMCs. (**A**) Volcano plot based on differentially expressed lncRNAs in PBMCs compared with healthy controls. (**B**) Volcano plot based on differentially expressed lncRNAs in PBMCs of co-culture compared with control. Volcano plot shows −log_10_ *p*-values on the *y*-axis and fold change expressed as log_2_ on the *x*-axis. Red scatter dots represent up-regulated lncRNAs with *p* ≤ 0.05 and log_2_fold change ≥ 1. Blue scatter dots represent down-regulated lncRNAs with *p* ≤ 0.05 and log_2_fold change ≤ −1. Green scatter dots represent a candidate of cancer-induced lncRNAs for validation in PBMCs. (**C**) The Venn diagram represents intersect genes between two transcription profiles of PBMCs including PBMCs from patients with HCC and PBMCs from a co-culture model. (**D**) Comparison of cancer-induced lncRNAs expression in PBMCs of patients with HCC and healthy controls. * *p* < 0.05, ** *p* < 0.01.

We performed a comparative analysis of lncRNA expression profiles from eight patients with HCC and four healthy controls to identify the lncRNA candidate biomarkers. The baseline characteristics of patients with HCC and healthy controls are presented in Supplementary Table S1. The differentially expressed lncRNAs (DElncRNAs) between PBMCs of patients with HCC and PBMCs of healthy controls were identified by cutting off at a log_2_fold-change of ≤−1 or ≥1, and *p* < 0.05 for each lncRNA, which included 298 up-regulated lncRNAs and 159 down-regulated lncRNAs (Figure 1A). To investigate the DElncRNAs of cancer-induced PBMCs, the data sets from PBMCs of three healthy controls co-cultured with HCC cells (Huh7) and without HCC cells were compared and analyzed. We found 114 up-regulated lncRNAs and 69 down-regulated in this co-culture model using the same mentioned cut-off (Figure 1B). The clustering of DElncRNAs was shown in heatmap plots. The expression profile showed a clear separation pattern between HCC and healthy controls (Figure S1A) as well as in co-culture model with HCC cells and without HCC cells (Figure S1B).

To identify whether cancer-induced lncRNAs from PBMCs could be biomarkers for HCC, the lncRNA profile of PBMCs from HCC was integrated with PBMCs from co-culture with HCC cells. The Venn diagram based on the intersection of DElncRNAs between the PBMCs from HCC and a co-cultured model is shown in Figure 1C. The lists of intersecting and individual profiles of DElncRNAs are present in Supplementary Table S2. In overlapping DElncRNAs of two profiles, we found three up-regulated lncRNAs, including *MIR4435-2HG*, *SNHG9* and *lnc-LCP2-1*, and one down-regulated lncRNAs including *lnc-POLD3-2*. The expression of these four DElncRNAs is shown in Figure 1D. To investigate the discrimination between patients with HCC and healthy controls based on these four lncRNAs, the expression of these lncRNAs was further analyzed by hierarchical clustering and is presented as a heatmap analysis, as shown in Figure S1C. This result demonstrated that these four lncRNAs clearly discriminated between HCC and healthy controls. Moreover, these four DElncRNAs in PBMCs were considered to be cancer-induced lncRNAs because of their alteration after inducing with HCC in the co-culture and also detected in the PBMCs of patients with HCC.

### 2.2. Prediction of Regulatory Network and Function Analysis of lncRNAs

To investigate the possible regulation role of lncRNAs and their targets via competing endogenous RNAs (ceRNAs) including miRNAs and mRNAs, the identified four cancer-induced lncRNAs were applied to predict miRNAs of lncRNA target based on shared miRNA recognition elements. Then, the 24 DEmRNAs of PBMCs were also used to predict miRNA–mRNA interaction. Together, the ceRNAs network was constructed by overlapping miRNA between lncRNA–miRNA and miRNA–mRNA interaction (Figure S2A). Finally, there were 139 interactions in the ceRNAs network, which included 4 lncRNAs, 62 miRNAs, and 23 mRNAs (Supplementary Table S3). This result demonstrated that these cancer-induced lncRNAs could play a role in mRNA regulation as miRNA sponges in PBMCs of HCC.

The functional enrichment analysis of the lncRNA target list was performed by TAM 2.0 to investigate the characteristics annotation of predicted lncRNA targets, including disease, functional annotation, and transcription factor (Figure S2B). The result of TAM2.0 is shown in Supplementary Table S4. The top 10 significantly enriched diseases were several types of cancers, which included HCC in the second of this term. Most of the significantly enriched functions were involved in cancer progression and immune response. In addition, we found that these lncRNA targets were related to the many transcription factors, which were associated with cancer and the immune response pathway, including TP53, MYC, and TGFβ. Together, these data represented that the regulation of four cancer-induced lncRNAs in PBMCs may be related to cancer and the immune system in HCC.

### 2.3. Validation of Candidate lncRNAs in Clinical Samples

From the above analyses, the four candidate lncRNAs (*MIR4435-2HG*, *SNHG9*, *lnc-LCP2-1*, and *lnc-POLD3-2*, green-labeled in Figure 1A,B) were selected to further investigate whether they might be novel diagnostic biomarkers for HCC at the cohort level. To this end, the expression of these lncRNAs was validated by the qRT-PCR method in PBMCs obtained from 100 patients with HBV-related HCC, 100 patients with chronic hepatitis B (CHB), and 100 healthy controls. The validated cohort was age- and gender-matched among the studied groups. The baseline characteristics of the patients in this validated cohort are shown in Table 1. It was shown that there were significant differences between the HCC and non-HCC groups in terms of biochemical parameters, including platelet count, serum albumin, aspartate aminotransferase (AST), alkaline phosphatase and AFP. Moreover, a higher proportion of cirrhosis was observed in the HCC group compared with the non-HCC group.

**Figure 2:**
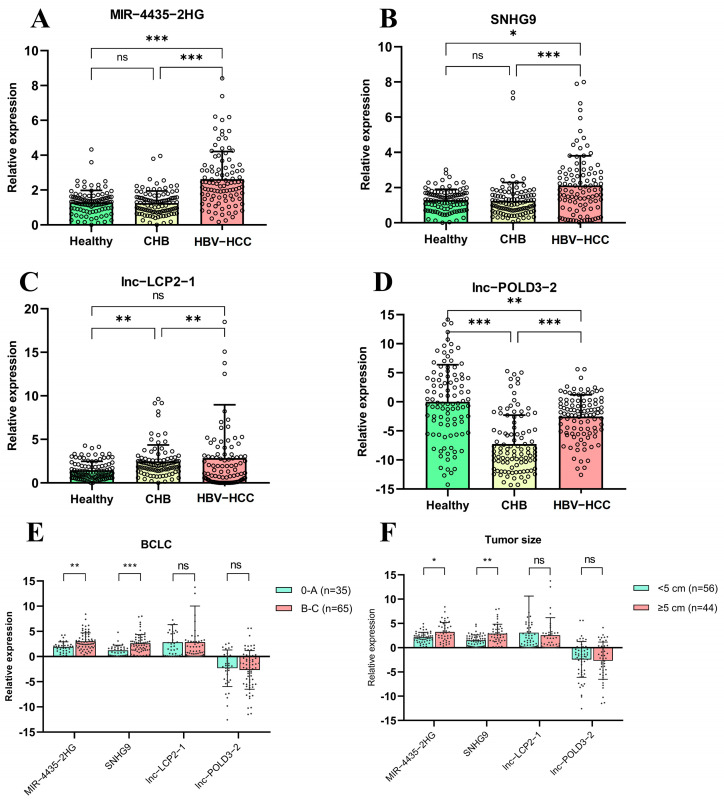
Relative expression of lncRNAs in PBMCs of healthy controls, patients with CHB and patients with HBV-HCC (**A**) *MIR4435-2HG*, (**B**) *SNHG9*, (**C**) *lnc-LCP2-1*, (**D**) *lnc-POLD3-2*, (**E**) Association of lncRNAs with BCLC stage (0-A vs. B-C) in patients with HCC, (**F**) Association of lncRNAs with tumor size (<5 cm vs. ≥5 cm.) in patients with HCC. * *p* < 0.05, ** *p* < 0.01, *** *p* < 0.001.

In this validation set, we found that the expressions of *MIR4435-2HG*, *SNHG9*, *lnc-LCP2-1*, and *lnc-POLD3-2* in the HCC group significantly differed from those of the other groups (Figure 2). Specifically, the relative expression of *MIR4435-2HG* was significantly higher in patients in the HCC group (2.63 ± 1.58) than in the non-HCC group (1.25 ± 0.69, *p* < 0.001) and healthy control group (1.30 ± 0.68, *p* < 0.001) (Figure 2A). The expression of *SNHG9* in patients with HCC (2.12 ± 1.68) was also significantly higher than that in the non-HCC group (1.24 ± 1.04, *p* < 0.001) and healthy control group (1.29 ± 0.61, *p* = 0.015) (Figure 2B). For *lnc-LCP2-1*, its level was significantly higher in patients with HCC (2.86 ± 6.12) than in the non-HCC group (2.48 ± 1.88, *p* = 0.006), but it did not significantly differ from healthy controls (1.45 ± 1.02, *p* = 0.716) (Figure 2C). In contrast, *lnc-POLD3-2* expression was significantly decreased in patients with HCC (−2.54 ± 3.75) than healthy controls (0.00 ± 6.39, *p* < 0.001), but it exhibited significantly higher expression when compared with the non-HCC group (−7.27 ± 4.98, *p* < 0.001) (Figure 2D).

### 2.4. Association of lncRNA Expressions with Clinical Parameters of HCC

To further investigate the association of lncRNAs with clinical parameters in patients with HCC, we demonstrated that the expressions of *MIR4435-2HG* and *SNHG9* were significantly lower in patients with early stages (BCLC 0-A) than in those with intermediate and advanced stages (BCLC B-C, *p* < 0.001) (Figure 2E). Similarly, *MIR4435-2HG* and *SNHG9* expressions were found to be significantly higher in patients with larger tumor size (≥5 cm) in comparison with smaller tumor size (<5 cm) (*p* = 0.005 and *p* < 0.001, respectively) (Figure 2F). Additionally, the expression of *SNHG9* was associated with AST (*p* < 0.001) and alanine transaminases (ALT, *p* = 0.048) (Figure S3). There was no significant correlation between the expression of lncRNAs and other clinical parameters.

### 2.5. The Expressions of lncRNAs as Diagnostic Markers for HCC

**Figure 3:**
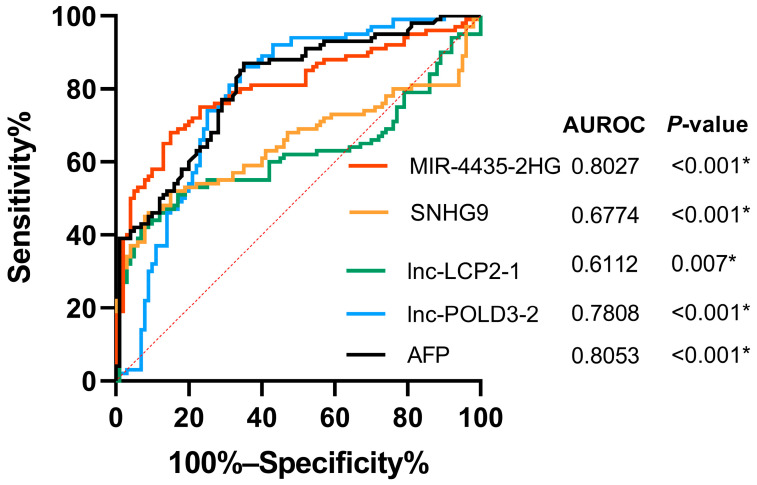
Area under receiver operating characteristic curves (AUROCs) of lncRNAs and AFP for differentiating the HCC group from the non-HCC group.

The receiver operating characteristic (ROC) was performed to investigate the diagnostic performance biomarkers for discriminating the HCC group from the non-HCC group (Figure 3). In this context, the area under ROC curve (AUROC) was 0.80 (95% confidence interval (CI); 0.74–0.87, *p* < 0.001) for *MIR4435-2HG*, 0.68 (95% CI 0.60–0.76, *p* < 0.001) for *SNHG9*, 0.61 (95% CI 0.71–0.85, *p* = 0.007) for *lnc-LCP2-1*, 0.78 (95% CI 0.30–0.46, *p* < 0.001) for *lnc-POLD3-2*, and 0.81 (95% CI 0.75–0.87, *p* < 0.001) for AFP.

Based on the ROC analysis, the optimal cut-off points of *MIR4435-2HG*, *SNHG9*, *lnc-LCP2-1*, and *lnc-POLD3-2* were 1.58, 1.36, 1.51 and −4.77, respectively. Details of the diagnostic performance of each lncRNA as well as AFP (cut-off 20 ng/mL) are shown in Table 2. Among them, *MIR4435-2HG* and *lnc-POLD3-2* exhibited better performance than *SNHG9* and *lnc-LCP2-1*. Additionally, the combined use of lncRNAs as diagnostic biomarkers was further evaluated (Supplementary Table S5). Again, the combination of *MI4435-2HG* and *lnc-POLD3-2* improved the sensitivity for detecting HCC without compromising their specificity as the AUROC reached 0.87.

The diagnostic role of *MI4435-2HG* and *lnc-POLD3-2*, as well as their combination, in patients with AFP-negative HCC (AFP < 20 ng/mL) and early-stage HCC was further investigated. In this study, there were 51 (51%) and 49 (49%) patients with AFP-negative and AFP-positive, respectively (Figure S4). Among the AFP-negative group, 74.5% (38/51) of patients had an elevated *MIR4435-2HG* level (≥1.8) and 75.5% (39/51) of patients had a high *lnc-POLD3-2* level (≥−4.8). For the AFP-positive group, high levels of *MIR4435-2HG* and *lnc-POLD3-2* were detected in 79.5% (39/49) and 73.5% (36/49), respectively. The combination of *MIR4435-2HG* and *lnc-POLD3-2* increased the detection of HCC to 88.2% (45/51) and 97.9% (48/49) in the AFP-negative and AFP-positive groups, respectively.

Among early HCC (stages 0-A), we found that 28.5% (10/35) of patients had elevated AFP concentration, while 62.9% (22/35) and 80.0% (28/35) of patients had elevated levels of *MIR4435-2HG* and *lnc-POLD3-2*, respectively (Figure S5). Moreover, the combination of *MIR4435-2HG* and *lnc-POLD3-2* increased the detection of early HCC to 85% (30/35). These data indicated that *MIR4435-2HG*, *lnc-POLD3-2* and their combination could be used as the potential biomarkers for detecting AFP-negative HCC and small HCC.

### 2.6. Prognostic Role of lncRNAs of Patients with HCC

**Figure 4:**
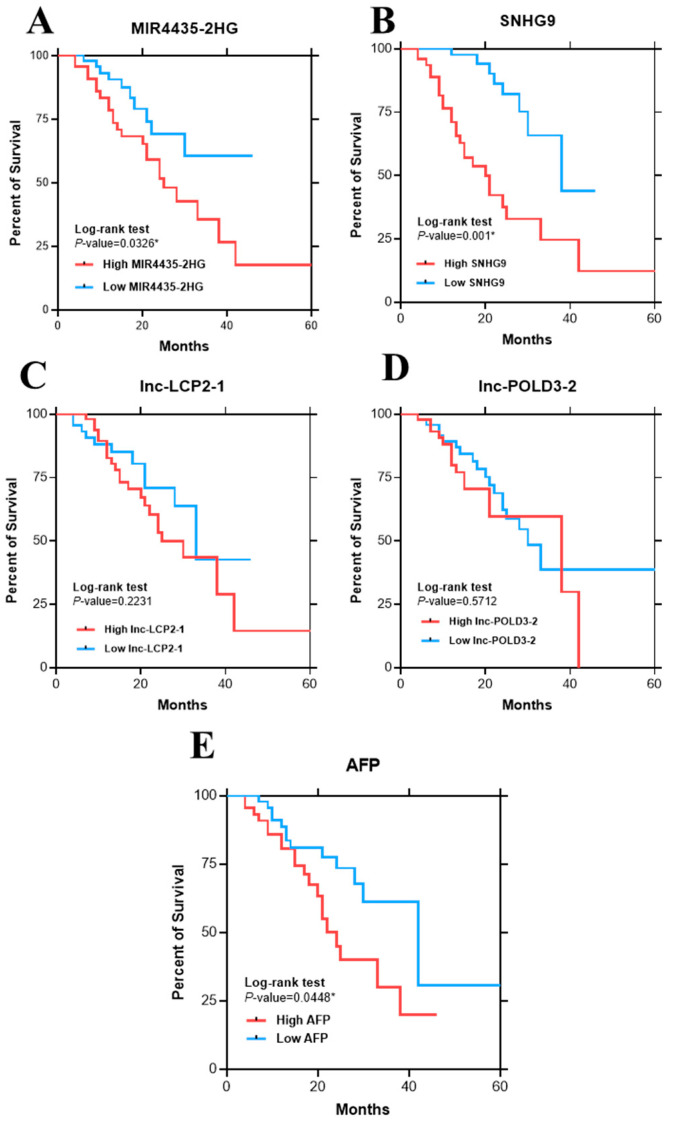
Kaplan–Meier survival curves for overall survival analysis of patients with HCC (**A**) *MIR4435-2HG* (**B**) *SNHG9* (**C**) *lnc-LCP2-1* (**D**) *lnc-POLD3-2* (**E**) AFP.

To investigate whether the expression of lncRNAs might be useful for predicting the overall survival of patients with HCC, the Kaplan–Meier analysis of each lncRNA using their median values as the cut-off points was performed. As shown in Figure 4A, a high expression of MI4435-2HG (≥2.4) was significantly associated with shorter overall survival compared with those of low expression (<2.4) (15 vs. 20 months, *p* = 0.033 by log-rank test). Similarly, a high expression of *SNHG9* (≥1.9) was significantly associated with shorter overall survival compared with patients with low expression (<1.9) (20 vs. 38 months, *p* = 0.001) (Figure 4B). However, the other lncRNAs were not associated with overall survival (Figure 4C,D). Regarding AFP, a high serum level (≥100 ng/mL) was significantly associated with poor prognostic compared to those with low serum level (<100 ng/mL) (24 vs. 42 months, *p* = 0.045) (Figure 4E).

All lncRNAs were further applied for Cox regression analysis together with other clinical parameters. In multivariate analysis, the presence of cirrhosis, advanced BCLC stages and elevated *SNHG9* expression were selected as independent predictive factors of overall survival (Table 3).

## 3. Discussion

Numerous studies support that lncRNAs are involved in many pathological processes, especially in cancer development [12]. The alteration of lncRNAs has been extensively investigated in hepatocarcinogenesis regarding the modulation of tumor proliferation and metastasis [13,14]. Interestingly, some cancer-related lncRNAs are expressed in body fluids, which opens up the possibility of using them as liquid biopsy for HCC diagnosis and prognosis [15]. The early diagnosis of HCC is extremely important, as it leads to an improved prognosis with the 5-year survival rates of 60–80% after curative treatments [2]. To date, the surveillance of HCC commonly relies on imaging findings and serum biomarkers. In a recent meta-analysis, the detection of early-stage HCC using ultrasound together with serum AFP has sensitivity of 60–70% [16]. However, the sensitivity of serum AFP alone in detecting early-stage HCC is approximately 50% [17], which was higher than our study (only 30%). The limitations of serum AFP emphasize the urgent need for identifying novel biomarkers to improve the early diagnosis of HCC.

There is increasing evidence that suggests that the alteration of lncRNAs in PBMCs could be used as a biomarker for diagnosis and disease monitoring in many chronic disorders [18,19,20]. Moreover, the transcriptomic analyses in PBMCs have revealed differential expression patterns between cancerous and non-cancerous samples in several types of malignant tumors, including HCC [21,22,23]. In this study, we demonstrated that DElncRNAs in PBMCs in the co-culture model could differentiate between HCC and non-HCC. Specifically, four DElncRNAs with three up-regulated and one down-regulated lncRNA were identified in HCC, including *MIR4435-2HG*, *SNHG9*, *lnc-LCP2-1*, and *lnc-POLD3-2*. It was previously revealed that the features of gene expression profiles in PBMCs significantly differed between patients with or without HCC and might be a surrogate marker representing tumor-infiltrating inflammatory cells in the liver [24]. Similarly, the profiling of PBMCs from a co-culture model with HCC cells was shown to be useful for predicting the risk of drug-induced liver injury, particularly among immune-related genes [25]. Recently, the concordance of transcriptomic profiles between PBMCs and tumor tissue-derived samples was also demonstrated in colorectal cancer [26]. These data suggest that the alteration of lncRNAs in PBMCs could be a readily accessible biomarker for tumor microenvironment in HCC.

The interaction between RNA molecules in HCC tissues was reported in many studies including miRNA–mRNA [27], lncRNA–mRNA [13], and miRNA–lncRNA [28] interaction. However, the regulatory complex of ceRNAs has not been investigated in PBMCs of HCC. Here, we constructed a ceRNAs network in PBMCs of patients with HCC that might explore a new role of lncRNAs in hepatocarcinogenesis. The ceRNAs network showed that *MIR4435-2HG* could play an essential role in HCC development by regulating the immune response gene via negative regulating of numerous miRNAs such as the *MIR4435-2HG*/*hsa-miR-1-3p*/*CXCL2* axis. Similar to our findings, a previous report demonstrated that the *MIR4435-2HG*/*hsa-miR-1-3p* axis was closely linked to immune cell infiltration in HCC [29]. Likewise, the interaction of *hsa-miR-1-3p*/*CXCL2* was associated with the immune mechanism of HCC development [30]. However, the actual mechanism by which these lncRNAs regulating hepatic carcinogenesis requires further validation in additional studies.

The functional analysis of cancer-induced lncRNAs targets was further elucidated. Our result showed that cancer-induced lncRNAs targets were involved with the numerous types of cancer including HCC. The enriched functions of lncRNAs targets consisted of cell proliferation, cell cycle, cell death, cell apoptosis, and immune response, which are directly related to the progression of cancers [31]. For instance, the dysregulation of lncRNAs can promote HCC growth, metastasis, and drug resistance via sponging other RNAs and interacting with proteins [32]. Regarding the regulation of lncRNAs in immune response, many lncRNAs are involved in immune checkpoint regulation and immunotherapy resistance [9]. Moreover, lncRNAs have been shown to be related to transcription factors such as *TP53* and *TGF-β*, which involve the transcription of many downstream genes and regulate the development of cancers [33,34].

Based on the ROC analysis, we subsequently found that only *MIR4435-2HG* and *lnc-POLD3-2* expression in PBMCs might be potential biomarkers for HCC. LncRNA *MIR4435-2HG*, located in human chromosome 2q13, was originally discovered as a GC-associated lncRNA [35]. It was reported that altered *MIR4435-2HG* influenced various biological processes including epithelial–mesenchymal transition (EMT), proliferation, apoptosis, migration and invasion [36]. In breast cancer cells, for example, recent evidence indicated that decreased *MIR4435-2HG* promoted apoptosis and suppressed cell proliferation and metastasis via inhibiting *Wnt*/*β-catenin* signaling [37]. Previously, the overexpression of *MIR4435-2HG* was correlated with the development of lung cancer, and the activity of β-catenin was up-regulated by the interaction between *MIR4435-2HG* and *β-catenin* [38]. Moreover, *MIR4435-2HG* was also recognized as a miRNA sponge of *TGF-β1* that accelerated the activation of *TGF-β* signaling [39]. In prostate cancer, the up-regulating *MIR4435-2HG* was correlated with elevated circulating *TGF-β1* and was associated with poor clinical and pathological features [40]. Together, these data indicate that *MIR4435-2HG* participates in *Wnt*/*β-catenin* and *TGF-β* pathways, which are well-established signaling that are involved in the pathogenesis of several cancers, including HCC [41].

Regarding HCC, many studies have reported that *MIR4435-2HG* was up-regulated in liver tissue and was associated with poor prognosis of patients with HCC [29,42,43]. Additionally, it was demonstrated that *MIR4435-2HG* played important roles in regulating miRNAs that led to the activation of several biological processes of HCC [42,43]. For instance, the overexpression of *MIR4435-2HG* induced HCC proliferation via the up-regulation of miRNA-487a [42]. In addition, it was shown in HCC cell lines that *MIR4435-2HG* facilitated tumor proliferation and metastasis by modulating miR-22-3p/YWHAZ signaling [43]. Unlike previous data obtained from liver tissue specimens, our study demonstrated that the up-regulated level of *MIR4435-2HG* in PBMCs was predictive of worse overall survival. Taken together, these data indicate that the up-regulated expression of *MIR4435-2HG* in liver tissue or PBMCs might be associated with the poor prognosis of patients with HCC.

In this study, we also demonstrated that circulating *lnc-POLD3-2* could be a novel diagnostic biomarker for discriminating HCC from non-HCC. As being demonstrated in our results, this lncRNA exhibited similar AUROC to that of AFP and *MIR4435-2HG.* Although the role of *lnc-POLD3-2* in hepatocarcinogenesis remains to be explored, the combination of this lncRNA and *MIR4435-2HG* was able to improve the performance for detecting early HCC. Additionally, circulating *MIR4435-2HG* and *lnc-POLD3-2* could be used to diagnose AFP-negative HCC. These results suggest the potential use of *MIR4435-2HG* and *lnc-POLD3-2* as novel sensitive biomarkers for early HCC detection as well as complementary tests in patients with HBV-related HCC who had low or normal levels of serum AFP.

Regarding the prognostic role of cancer-induced lncRNAs, the Kaplan–Meier analysis demonstrated that a high expression of circulating *MIR4435-2HG* and *SNHG9* was significantly associated with shorter overall survival in patients with HCC. These results might reflect the positive correlation of these lncRNAs expression with increased tumor size and advanced BCLC stage. However, the Cox regression analysis showed that only *SNHG9* was selected as an independent predictor of overall survival, while other blood-based biomarkers did not reach the significance in multivariate analysis. This finding suggests that circulating *SNHG9* could be a novel prognostic marker for HBV-related HCC. *SNHG9* (Small nucleolar RNA host gene 9) has been reported as a promising oncogenic lncRNA in various cancers [44]. A previous study demonstrated that up-regulating *SNHG9* was associated with poor prognosis in glioblastoma, as this lncRNA promoted tumor cell proliferation via regulating *Wnt2* and *miR-199a-5p* [45]. In lung cancer, *SNHG9* played a role in cisplatin resistance by promoting the *CAPRIN1* gene [46], and the knockdown of *SNHG9* decreased cell proliferation and invasion [47]. With regard to liver cancers, it was shown that *SNHG9* promoted hepatoblastoma tumorigenesis via *miR-23a-5p*/*Wnt3a* axis [48]. Moreover, decreasing *SNHG9* reduced methylation of the *GSTP1* gene and inhibited liver cancer cell proliferation, migration and invasion [49].

This study has certain limitations, as the number of clinical samples was relatively small. Moreover, our study focused on patients with chronic HBV infection; thus, the results might not be directly applicable to other etiological causes of HCC, such as chronic HCV infection and fatty liver disease. Additionally, we used only the Huh7 cell line in the co-culture experiments. Thus, it would be interesting to investigate the alteration of lncRNAs in HBV-related cell lines such as Hep3B and PLC/PRF/5 in further studies. To our knowledge, this is the first study of transcriptomic analysis in PBMCs and a co-culture model of HCC, which might possibly reflect cancer-induced lncRNAs in PBMCs. In summary, our data demonstrated that circulating *MIR4435-2HG* and *lnc-POLD3-2* either alone or in combination could be used as sensitive biomarkers of HCC detection in patients with chronic HBV infection. Apart from the diagnostic role, elevated *SNHG9* expression was shown to be an independent prognosis factor for patients with HCC. Considering these data, lncRNAs in PBMCs could potentially serve as novel biomarkers for HCC diagnosis and prognosis. Further studies are worthwhile to confirm our observations as well as to elucidate the mechanisms and clinical significance of these lncRNAs in patients with HCC.

## 4. Materials and Methods

### 4.1. Specimen Collection

Blood specimens were collected in an EDTA tube from patients with HBV–HCC before treatment with Transarterial Chemoembolization (TACE). Patients were diagnosed and treated at King Chulalongkorn Memorial Hospital, Bangkok, Thailand between 2018 and 2020. The imaging studies results of dynamic computed tomography (CT) or magnetic resonance imaging (MRI) were used for HCC diagnostic according to the American Association for the Study of Liver Diseases (AASLD) guideline. The demographic and clinical characteristics including gender, age, liver function tests, serum AFP level and HCC staging classified by the Barcelona Clinic Liver Cancer (BCLC) system of patients were obtained. The blood specimens were also collected from healthy controls and chronic HBV-infected patients without evidence of HCC as control groups in this study.

### 4.2. PBMCs Preparation

PBMCs were isolated from 6 mL of fresh EDTA blood specimens of patients with HCC prior to treatment using Percoll PLUS density gradient media according to the manufacturer’s specifications (GE Healthcare). Briefly, PBMCs were isolated at 2800 rpm for 20 min at room temperature and were then washed 2 times with PBS. Isolated PBMCs were suspended in 10% DMSO and fetal–bovine serum (FBS) and stored at −80 °C until further experiment.

### 4.3. Cell Culture

Liver cancer cells (Huh7, JCRB0403) were purchased by the National Institutes of Biomedical Innovation, Health, and Nutrition JCRB Cell Bank (Osaka, Japan). Huh7 cells were maintained in DMEM medium (Gibco) and supplemented with 10% FBS at 37 °C 5% CO_2_. The cells with 80% confluence were dissociated using 0.05% Trypsin with 0.5 mM EDTA and were then washed by using phosphate buffer saline (PBS). Three healthy individual PBMCs were co-cultured with Huh7 cells in Transwell culture six-well plates with 0.4 μm pore size (Costar) for the analysis of the cancer-induced lncRNAs in PBMCs by liver cancer cells. Briefly, Huh7 cells (10^6^ cells) were seeded into a lower chamber with DMEM and were then incubated overnight at 37 °C and 5% CO_2_. PBMCs from 3 healthy individuals (2 × 10^6^ cells) were individually plated into an upper chamber and incubated for 4 h at 37 °C and 5% CO_2_. Then, cancer-induced PBMCs were collected and performed RNA extraction.

### 4.4. RNA Preparation and RNA-Sequencing

The total RNA was extracted from PBMCs using TRIzol reagent (Gibco) according to the manufacturer’s instructions. Before RNA sequencing, the quality of RNA was accessed including a concentration of total RNA samples using a Qubit RNA assay kit (Invitrogen) and RNA integrity using RNA Electrophoresis with the 2100 Bioanalyzer System (Agilent). Then, library preparation and the sequencing of RNA samples of good quality (total RNA > 1 μg and RNA integrity > 7) were performed by Vishuo Biomedical (Vishuo Biomedical, Singapore). Briefly, library preparation was performed using a NEBNext Ultra RNA Library Prep Kit (NEB) according to Illumina RNA library preparation protocol. RNA was captured and isolated by the NEBNext RNA Magnetic Isolation Module (NEB). Double-strand cDNA was synthesized using random primer, ProtoScript II Reverse Transcriptase and Second-strand synthesis Enzyme mix. Then, both ends of cDNA were repaired and received a poly A-tail, which were followed by a T-A ligation to adding adaptors at both ends of cDNA. Each cDNA was amplified by PCR for 11 cycles using universal P5 and P7 primers. The product length and PCR were performed by a 2100 Bioanalyzer and evaluated by Qubit 2.0 Fluorometer. Library reads were then multiplexed and loaded on an Illumina HiSeq sequencer (Illumina). Sequencing data of 2 × 150 bp paired end was carried out. Ultimately, we obtained an average of 25 million read pairs per sample, which ranged from 20 to 37 million reads. The proportion of quality read above Q30 was more than 90% of the total reads for each sample.

### 4.5. Data Processing and Analysis

The sequencing quality control was conducted by FasTQC version 0.11.9 to check the overall quality [50]. The paired-end raw reads were trimmed by Fastp version 0.21.1 to remove the sequencing adaptor and filter out the low-quality sequences (less than Q30) [51]. The trimmed reads were mapped with the long non-coding RNA transcript sequences (GENCODE version 40) of *Homo sapiens* reference genome (GRCh38) using HISAT2 version 2.2.1 [52]. Mapped reads were assembled into transcripts by StringTie version 2.1.6 for the lncRNA assembly and quantification [53]. The information of mapped reads analysis is presented in Supplementary Table S6. Then, the differential expression of lncRNAs was performed by DESeq2 version 1.34 to compare lncRNA expression between PBMCs from patients with HCC and healthy controls as well as PBMCs from co-culture with HCC and without HCC [54]. Long non-coding RNAs with a read count ≥ 20 reads in ≥5 samples were retained for further analysis. The up-regulated and down-regulated lncRNAs were identified by setting a cut-off at log_2_fold change ≥1 or ≤−1 and *p*-value ≤ 0.05 as DElncRNAs [55].

Intersected DElncRNAs from PBMCs and the co-culture model were used to predict lncRNA–miRNA interaction using mirNET version 2.0 and lncBase version 3 with the default setting [56,57]. The DEGs from our previous study were also used for predicting miRNA–mRNA interaction using mirNET. Using the previous prediction, the regulatory network was built by the overlapping of miRNAs between lncRNA–miRNA prediction and miRNA–mRNA prediction in accordance with the ceRNAs hypothesis. This ceRNAs network was visualized using the Cytoscape version 3.9.1 [58]. Then, the lncRNA–target miRNAs enrichment analysis was applied using TAM2.0 to explore the main function and associated disease [59].

### 4.6. Quantification RT-PCR for lncRNAs Validation

The DElncRNAs were validated in PBMCs of patients with HBV-related HCC and non-HCC individuals by qRT-PCR. In this, *MIR4435-2HG*, *SNHG9*, *lnc-LCP2-1*, *and lnc-POLD3-2* were selected for validation by overlapping DElncRNAs from PBMCs of patients and PBMCs of co-culture with HCC cells. The total RNA was extracted from PBMCs of 100 patients with HBV-related HCC, 100 patients with chronic hepatitis B and 100 healthy controls using TRIzol reagent (Thermo Scientific, Carlsbad, CA, USA) and was then treated with DNase I (Thermo Scientific, Carlsbad, CA, USA) to digest DNA in extracted RNA. After that, cDNA was synthesized using RevertAid First Strand cDNA Synthesis (Thermo Scientific, Carlsbad, CA, USA). The qRT-PCR reaction included 6.25 µL of QPCR Green Master Mix HRox 2 × (Biotechrabbit, Berlin, Germany0.25 μL of primers and 1 μL of cDNA and nuclease-free water in a total volume of 12.5 μL. The reactions were carried out on a QuantStudio 5 Real-Time PCR System (Applied Biosystems, Carlsbad, CA, USA). Primers and thermal cycle information are shown in Supplementary Table S7. The analysis of qRT-PCR was performed by a duplicate of each sample with positive controls for each target gene and negative controls for interpretation. The expression of target lncRNAs was normalized by GAPDH endogenous reference gene. The expression data are shown in log_2_fold change format.

### 4.7. Statistical Analysis

Statistical analysis was performed using the Statistical Package for the Social Sciences (SPSS) version 23 (https://www.ibm.com/analytics/spss-statistics-software, accessed on 13 March 2022) and GraphPad prism version 9 for Windows (https://www.graphpad.com/scientific-software/prism, accessed on 13 March 2022). Comparisons between groups were analyzed by Chi’s square or Fisher’s exact test for categorical variables and by Student’s *t*-test or one-way ANOVA for continuous variables. Spearman’s rank test was used for correlations between parameters. Kaplan–Meier analysis and a log-rank test were used for survival analysis. The Cox regression analysis was conducted to identify independent factors associated with the overall survival of patients with HCC. A *p*-value < 0.05 was considered statistically significant. The adjusted *p*-value for the multiple hypothesis testing was not performed in this study due to the small number of samples. However, we further validate the results with a highly sensitive method using qPCR in the independent cohort.

## Figures and Tables

**Table 1:** Baseline characteristic of patients in the validated cohort.

Baseline Characteristics	Healthy Controls	Patients without HCC	Patients with HCC	*p*
	(n = 100)	(n = 100)	(n = 100)	
Age (years)	56.3 ± 3.4	56.7 ± 8.4	57.7 ± 8.6	0.053
Gender (Male)	80 (80.0)	85 (85.0)	85 (85.0)	0.549
Total bilirubin (mg/dL)		0.9 ± 0.7	1.0 ± 0.7	0.729
Serum albumin (g/dL)		4.1 ± 0.6	3.6 ± 0.5	<0.001 *
Aspartate aminotransferase (IU/L)		36.6 ± 30.2	64.5 ± 53.5	<0.001 *
Alanine aminotransferase (IU/L)		42.9 ± 42.3	44.8 ± 25.8	0.709
Alkaline phosphatase (IU/L)		77.3 ± 50.6	125.3 ± 70.8	<0.001 *
Platelet count (10^9^/L)		223.2 ± 74.6	176.0 ± 93.9	0.002 *
Alpha fetoprotein (ng/mL)		8.5 ± 13.9	6928.1 ± 28,444.2	0.016 *
Presence of cirrhosis		11 (11.0)	80 (80.0)	<0.001 *
BCLC stage (0-A/B/C)		-	35 (35.0)/49 (49.0)/15 (15.0)	-

Data expressed as mean ± SD, BCLC; Barcelona Clinic Liver Cancer, * *p*-value < 0.05.

**Table 2:** Summary of diagnostic performance of lncRNAs.

Marker	AUROC	Sensitivity (%)	Specificity (%)	PPV (%)	NPV (%)	Accuracy (%)	Cut-Off	95% CI	*p*
MIR-4435-2HG	0.80	75.00	75.00	75.00	75.00	75.00	1.5781	0.74–0.87	<0.001 *
*SNHG9*	0.68	66.00	67.00	66.67	66.34	66.50	1.3579	0.60–0.76	<0.001 *
*lnc-LCP2-1*	0.61	64.29	42.64	37.82	68.75	50.25	1.5105	0.71–0.85	0.007 *
*lnc-POLD3-2*	0.78	74.00	75.00	74.75	74.26	74.50	−4.7687	0.30–0.46	<0.001 *
AFP (ng/mL)	0.81	48.00	88.00	80.33	62.86	68.16	20.0000	0.75–0.87	<0.001 *

PPV = Positive predictive value, NPV = Negative predictive value, CI = Confidence interval, * p-value < 0.05.

**Table 3:** Variables associated with overall survival in patients with HCC.

Variables	Category	Overall Survival			
		Univariate Analysis		Multivariate Analysis	
		OR (95% CI)	*p*	OR (95% CI)	*p*
Age (years)	<60 vs. ≥60	0.967 (0.49–1.92)	0.925		
Gender	Male vs. Female	1.69 (0.69–4.14)	0.250		
Total bilirubin (mg/dL)	<1.2 vs. ≥1.2	1.27 (0.61–2.68)	0.523		
Serum albumin (g/dL)	<3.5 vs. ≥3.5	0.45 (0.22–0.93)	0.031 *	1.31 (0.48–3.59)	0.598
Aspartate aminotransferase (IU/L)	<60 vs. ≥60	2.86 (1.44–5.68)	0.003 *	2.04 (0.77–5.44)	0.154
Alanine aminotransferase (IU/L)	<50 vs. ≥50	1.95 (0.94–4.06)	0.071		
Platelet count (10^9^/L)	≥100 vs. <100	1.41 (0.58–3.42)	0.446		
Presence of cirrhosis	No vs. Yes	0.34 (0.19–0.77)	0.008 *	0.25 (0.84–0.73)	0.011 *
Alpha fetoprotein (ng/mL)	<100 vs. ≥100	2.76 (1.34–5.67)	0.006 *	2.13 (0.78–5.82)	0.140
Tumor size (cm)	<5.0 vs. ≥5.0	3.32 (1.57–7.01)	0.020 *	0.64 (0.19–2.21)	0.481
BCLC stage	0-A vs. B-C	4.30 (2.47–7.47)	0.001 *	5.54 (1.66–46.16)	0.015 *
*MIR4435-2HG*	<2.4 vs. ≥2.4	2.20 (1.04–4.66)	0.039 *	0.75 (0.29–1.97)	0.560
*SNHG9*	<1.9 vs. ≥1.9	4.81 (2.16–10.71)	0.001 *	3.25 (1.40–7.53)	0.006 *
*lnc-LCP2-1*	<1.1 vs. ≥1.1	1.56 (0.75–3.22)	0.231		
*lnc-POLD3-2*	<2.0 vs. ≥2.0	1.22 (0.61–2.44)	0.575		

* *p*-value < 0.05.

## Data Availability

All sequencing data generated in this study are available at NCBI SRA database under Bioproject number PRJNA717231.

## Supplementary Materials

The following supporting information can be downloaded at: https://www.mdpi.com/article/10.3390/ijms23147882/s1.
