# Supplementary figures and images for: Transcriptomic Analyses Reveal Long Non-Coding RNA in Peripheral Blood Mononuclear Cells as a Novel Biomarker for Diagnosis and Prognosis of Hepatocellular Carcinoma
Source: Int J Mol Sci. 2022 Jul 17;23(14):7882. doi: 10.3390/ijms23147882 (PMC9324406; doi:10.3390/ijms23147882)

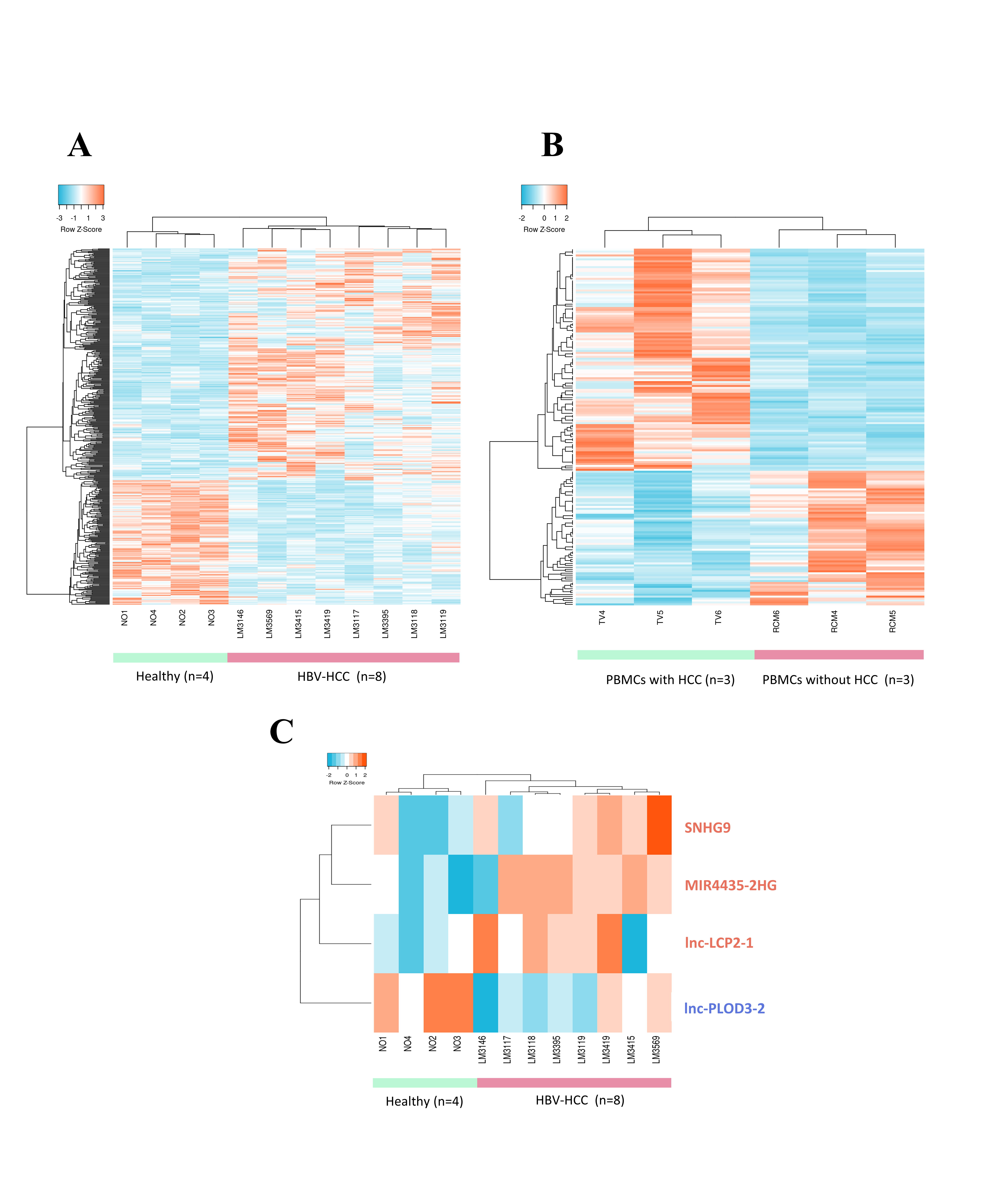

Supplement: Supplementary file 1 [file ijms-23-07882-s001.zip › Figure S1 Heatmap of cancer-induced lncRNA expression.tif]

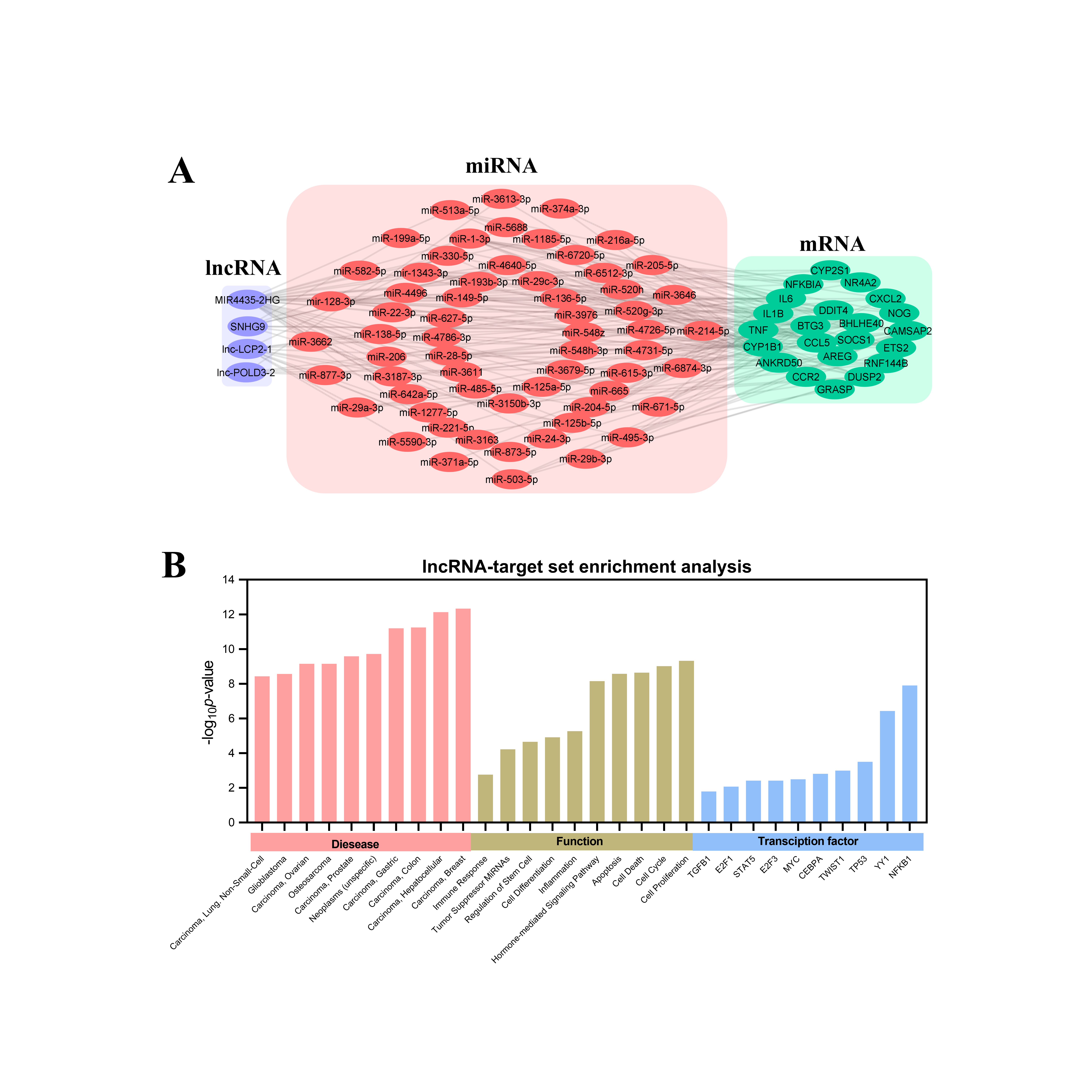

Supplement: Supplementary file 1 [file ijms-23-07882-s001.zip › Figure S2 lncRNA-target network and functional analysis (A) Predicted Competitor endogenous RNAs (ceRNAs) network (B) Top 10 lncRNA targets enrichment analysis using TAM2.0.tif]

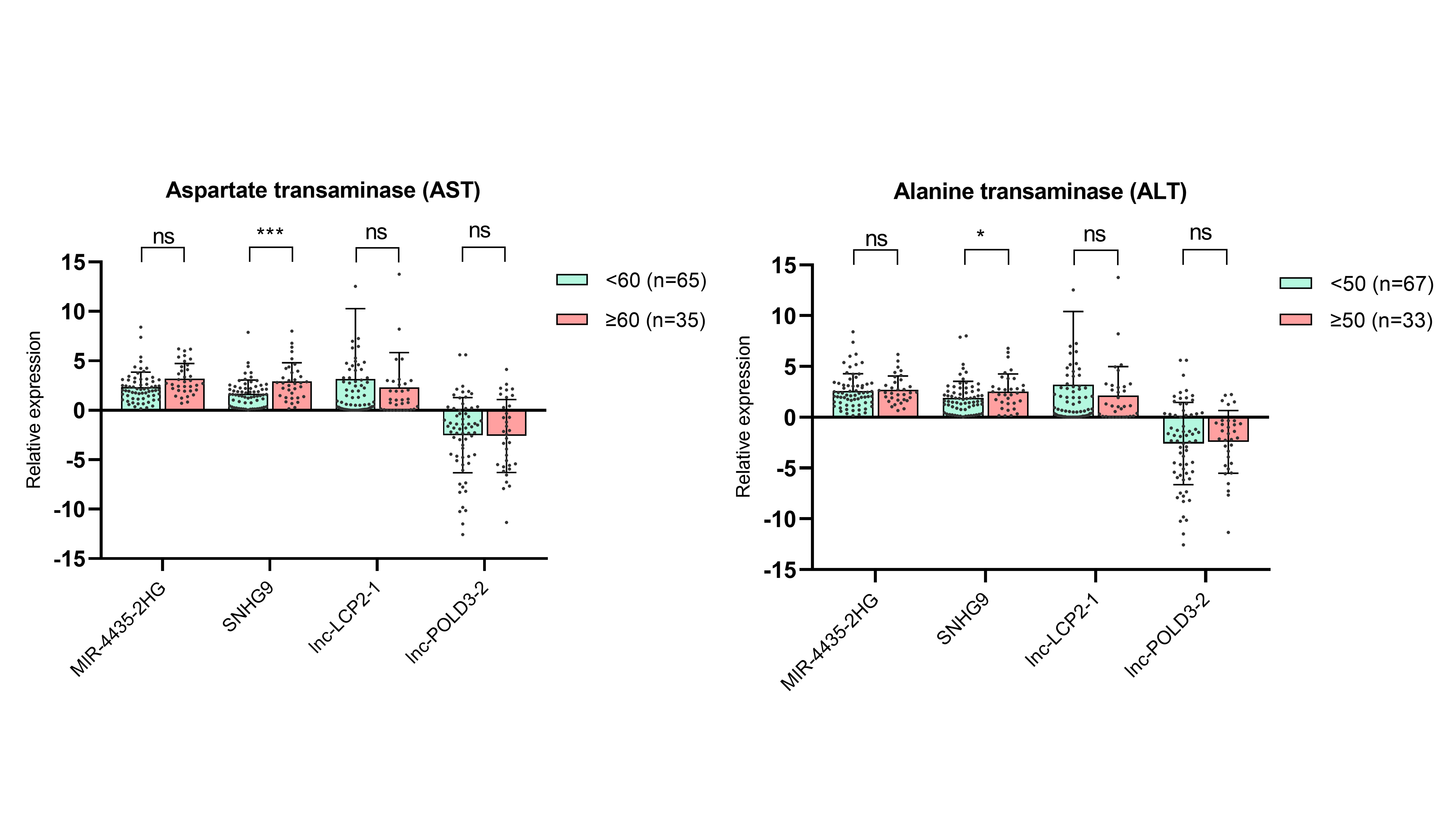

Supplement: Supplementary file 1 [file ijms-23-07882-s001.zip › Figure S3 Association of cancer-induced lncRNAs and liver enzyme.tif]

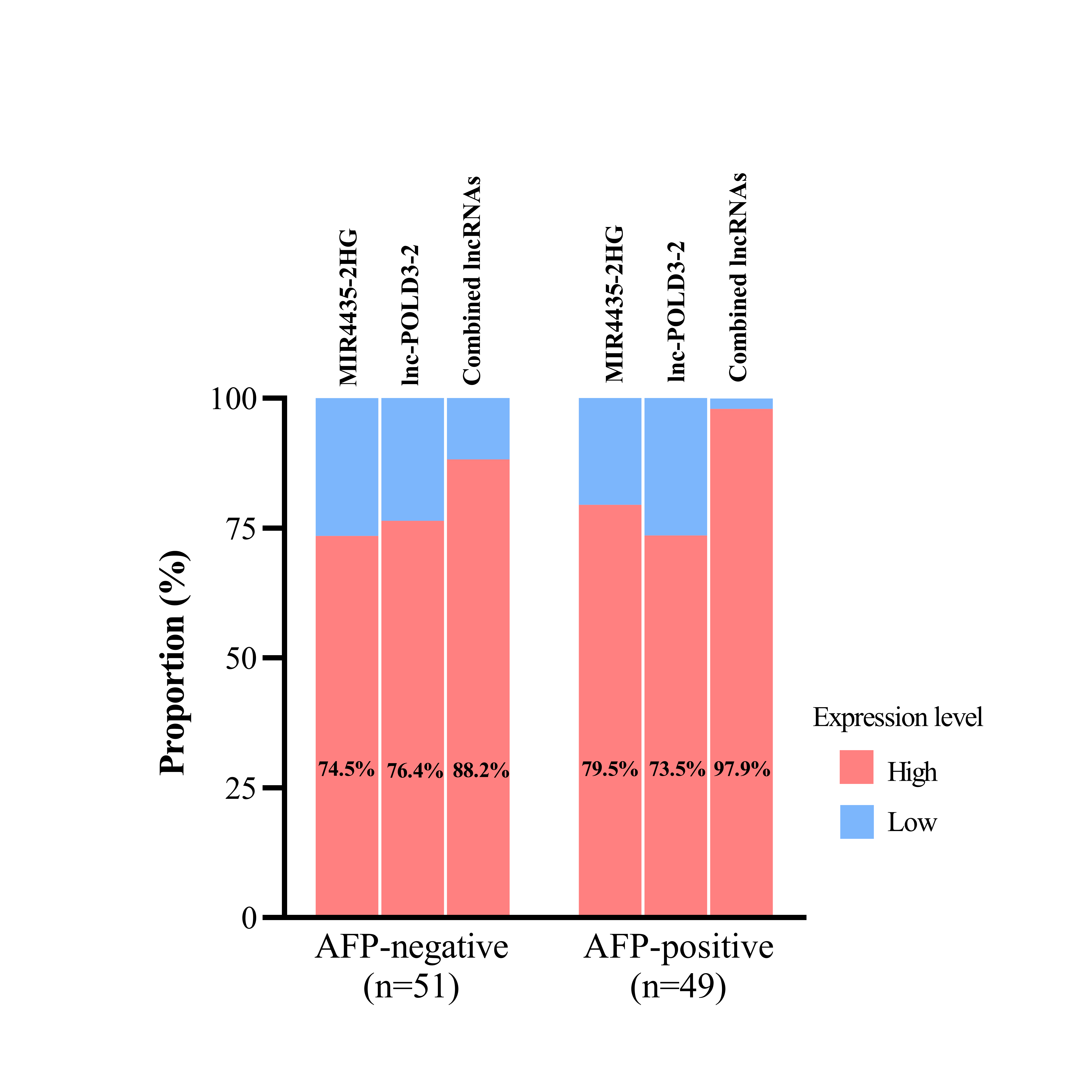

Supplement: Supplementary file 1 [file ijms-23-07882-s001.zip › Figure S4 Proportion of MIR4435-2HG and lnc-POLD3-2 expression in PBMCs of AFP- and AFP+ patients with HBV-HCC.tif]

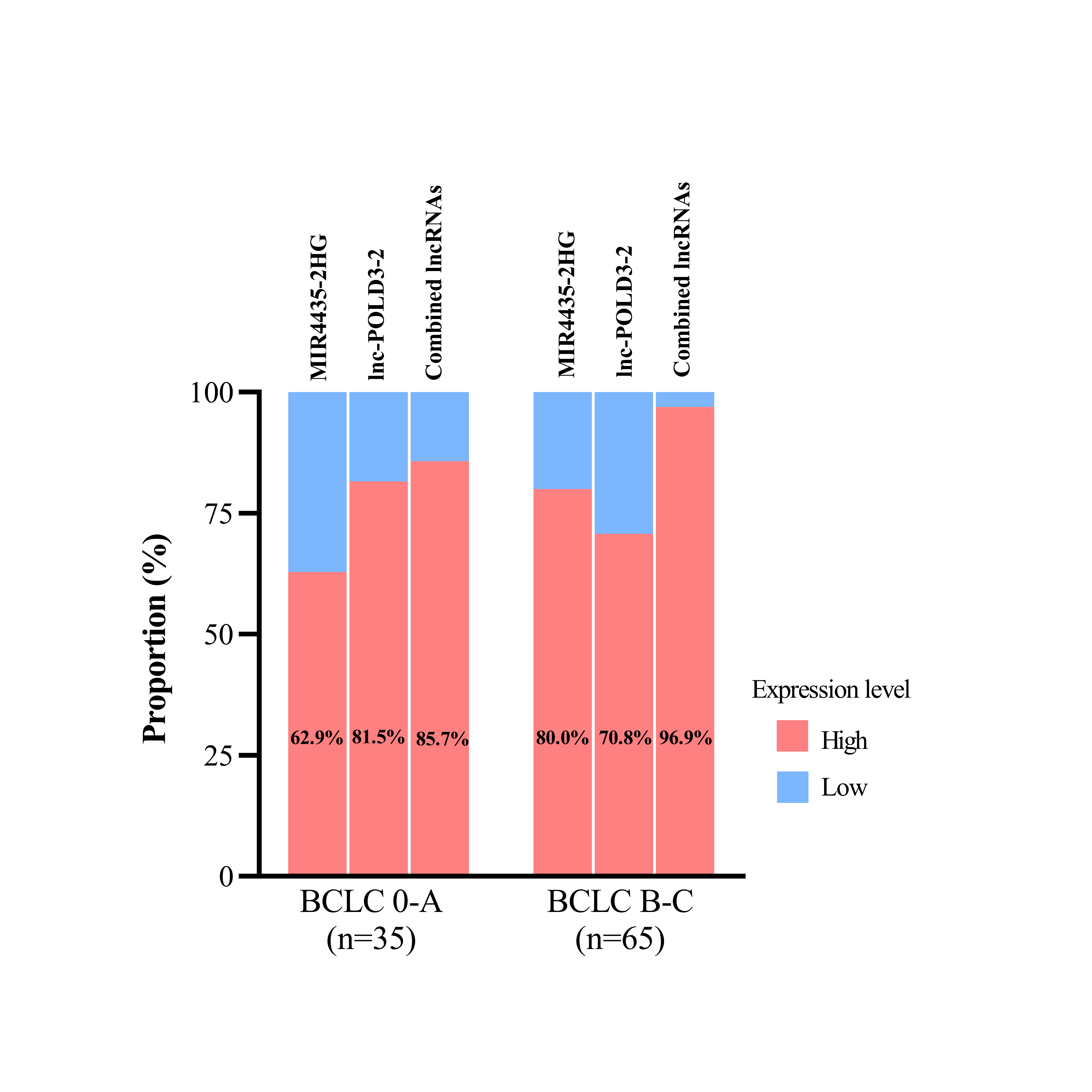

Supplement: Supplementary file 1 [file ijms-23-07882-s001.zip › Figure S5 Proportion of MIR4435-2HG and lnc-POLD3-2 expression in PBMCs of BCLC 0-A stage and B-C stage patients with HBV-HCC.tif]
